# A Role for Drosophila dFoxO and dFoxO 5′UTR Internal Ribosomal Entry Sites during Fasting

**DOI:** 10.1371/journal.pone.0011521

**Published:** 2010-07-09

**Authors:** Eugenia Villa-Cuesta, Brian T. Sage, Marc Tatar

**Affiliations:** Department of Ecology and Evolutionary Biology, Brown University, Providence, Rhode Island, United States of America; University of Washington, United States of America

## Abstract

One way animals may cope with nutrient deprivation is to broadly repress translation by inhibiting 5′-cap initiation. However, under these conditions specific proteins remain essential to survival during fasting. Such peptides may be translated through initiation at 5′UTR Internal Ribosome Entry Sites (IRES). Here we show that the *Drosophila melanogaster* Forkhead box type O (dFoxO) transcription factor is required for adult survival during fasting, and that the 5′UTR of *dfoxO* has the ability to initiate IRES-mediated translation in cell culture. Previous work has shown that insulin negatively regulates dFoxO through AKT-mediated phosphorylation while dFoxO itself induces transcription of the insulin receptor *dInR*, which also harbors IRES. Here we report that IRES-mediated translation of both dFoxO and dInR is activated in fasted Drosophila S2 cells at a time when cap-dependent translation is reduced. IRES mediated translation of dFoxO and dInR may be essential to ensure function and sensitivity of the insulin signaling pathway during fasting.

## Introduction

Changes in nutrient availability produce contrasting physiological states of fasting and feeding. This variation in nutrition is managed at both cellular and systemic levels. One cellular response is to repress the overall level of 5′cap-initiated translation [1]–[4] as seen in glucose deprived yeast [5], fasted rats [6], [7], and amino acid deprivation in many organisms (reviewed in [8]). Repressing the overall production of proteins is likely to reduce metabolite demands when nutrients are limited, but the synthesis of some proteins is essential for cells to positively adapt to the stress [3].

When deprived of glucose, yeast cells appear to maintain some proteins by translation initiation at Internal Ribosomal Entry Sites (IRES) within the 5′ UTR [5]. While IRES are best described in viral mRNA [9], [10], cellular IRES have been described in a limited but increasing number of mRNAs [1], [11] [See IRES database (http://ifr31w3.toulouse.inserm.fr/iresdatabase/]. The function of IRES in these cellular mRNAs has remained elusive, but their distribution among gene types suggests that IRES-dependent translation allows physiological adaptation to stress [5].

Several genes of the fly *Drosophila melanogaster* have identified IRES, including the insulin-like receptor *dInR* [12]. Importantly, the insulin receptor is at the interface between the systemic and cellular response to nutrients [13]–[16]. As conserved from mammals to Drosophila [17], fasting reduces the circulating level of insulin. Reduced binding of insulin at the insulin-like receptor inhibits the serine-threonine kinase AKT, blocking phosphorylation of dFoxO. dFoxO may then be transported to the nucleus to induce many genes associated with fasting metabolism. Several Drosophila studies suggest dFoxO is an important transcription factor involved in the adaptation to nutrient stress [15], [18]. Global gene expression profiling showed that 28% of genes up-regulated during nutrient deprivation are also induced by expression of constitutively active dFoxO in Drosophila S2 cells [18]. Among these genes are the insulin-like receptor *dInR* [15], and the translation regulator *4E-BP* [19], [20], [21]. This reciprocal connection between dInR post-translational regulation of dFoxO and dFoxO transcriptional control of *dInR* suggests that this signaling pathway functions as a homeostatic feedback network. It will increase insulin sensitivity by increasing receptor abundance in cells of peripheral tissues when circulating nutrients are low, and decrease insulin sensitivity when nutrients are high.

Here we investigate the role IRES play in the maintenance of the insulin signal transduction pathway during fasting. We show that during fasting, when general levels of translation are reduced, the relative abundance of dFoxO protein increases, and that this increase occurs without changing the level of *dfoxO* mRNA. We find that the 5′UTR of *dfoxO* has the ability to initiate IRES-mediated translation, and that *dfoxO*-IRES translation is active when fasting decreases cap-dependent translation. Furthermore, translation is also initiated at the IRES of *dInR* in fasting cells. Cap-independent translational control of dFoxO and of dInR may be a molecular mechanism that allows fasting cells to acquire an insulin-sensitive state and thus adapt the cells to respond rapidly to re-feeding.

## Results

### dFoxO confers survival during fasting

**Figure 1:**
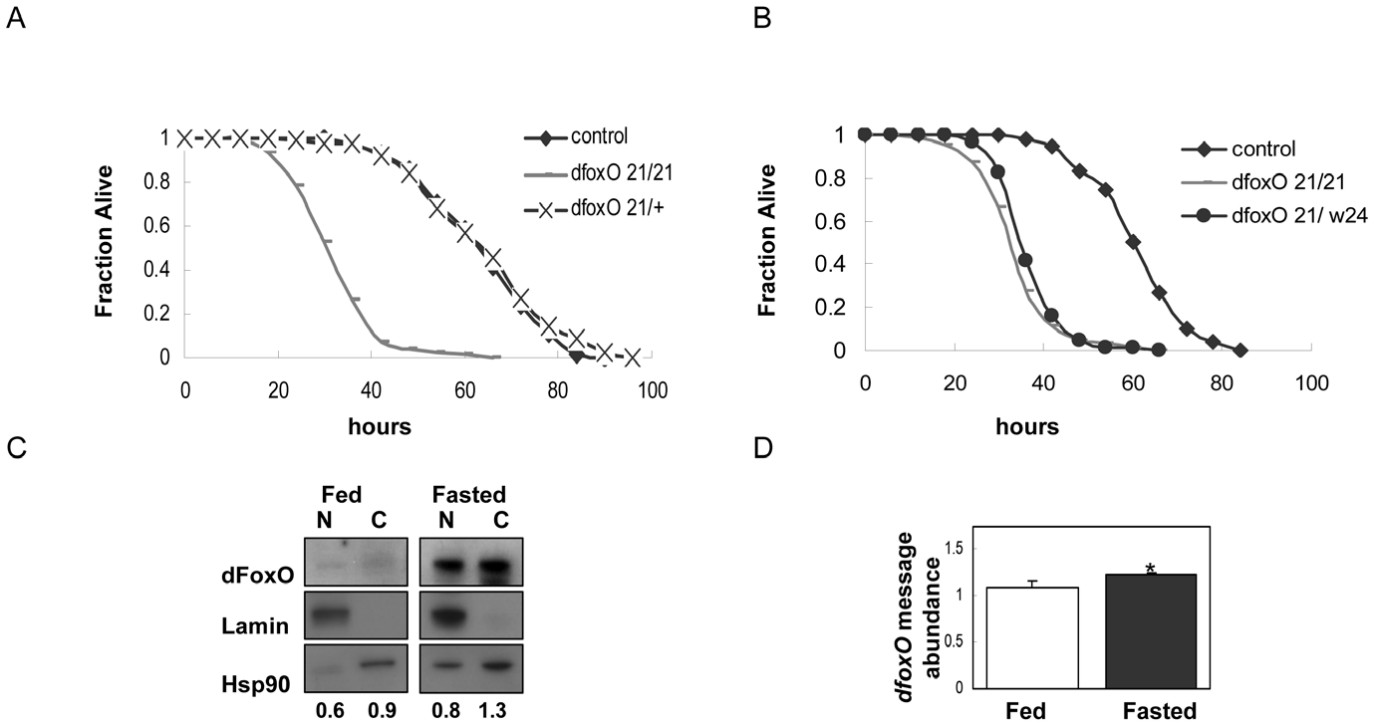
*Relative dFoxO* protein increases and confers survival during fasting. Null mutants homozygous (**A**) *dfoxO^21^* and (**B**) trans-heterozygous *dfoxO^21^*/*dfoxO^w24^* have reduced survival during fasting relative to wild type and *dfoxO^21^* ^/+^. (**C**) Sub-cellular localization of dFoxO in decapitated bodies of fed and fasted females. Lysates were analyzed by Western immunoblot for dFoxO, Lamin as nuclear (N) and Hsp90 as cytosolic (C) controls. Numbers indicate the quantification by densitometry to obtain the ratio dFoxO to Lamin in N, or Hsp90 in C. dFoxO nuclear quantification was corrected for contamination by subtracting Hsp90 from nuclear fractions. Likewise, Lamin in the cytosol was subtracted from the dFoxO cytosolic quantifications. The nuclear fraction lane from the fasted samples shows a prominent Hsp90 band that we have considered contamination. However, Hsp90 may translocate to the nucleus upon fasting. In that situation, the ratio dFoxO to Lamin would be higher that 0.8. (**D**) *dfoxO* mRNA abundance from decapitated bodies of fed and fasted females flies. Relative gene expression was analyzed by 2^−ΔΔCT^ method [33]. Data are shown as the mean +1 standard deviation. *, P<0.05 versus control.

Overexpression of dFoxO in larvae slows growth, inhibits feeding and produces small adults [22]. In adults the role of dFoxO during fasting is ambiguous. Loss of *dfoxO* reduces the capacity to survive upon amino acid depleted diet [23] although an independent study found *dfoxO* null flies were insensitive to starvation when measured on the scale of days [24]. To clarify the role of *dfoxO* during fasting, we studied the survival time of various homozygous null *dfoxO* flies maintained on media without yeast and sugar. The survival time of null homozygotes (*dfoxO^21/21^*) but not of heterozygotes (*dfoxO^21/+^*) was reduced by 50% on the nutrient depleted media (Fig. 1A). Null flies of the t*rans*-genotype *dfoxO^21^*/*dfoxO^w24^* were likewise sensitive to total nutrient deprivation (Fig. 1B).

### Relative dFoxO protein increases during fasting

FoxO proteins are post-translationally modified to regulate their localization in response to nutrition [25]. To study dFoxO protein cellular distribution in fasted flies, we examined its localization by western blot analysis of cell fractionated samples from body tissue. Relative to Lamin and Hsp90 controls, levels of dFoxO were elevated in both cytosolic and nuclear compartments in the bodies of fasted flies (Fig. 1C). Additionally, there was a small increase in the level of *dfoxo* transcripts (Fig. 1D).

### *dfoxO* 5′UTR can initiate IRES-mediated translation

**Figure 2:**
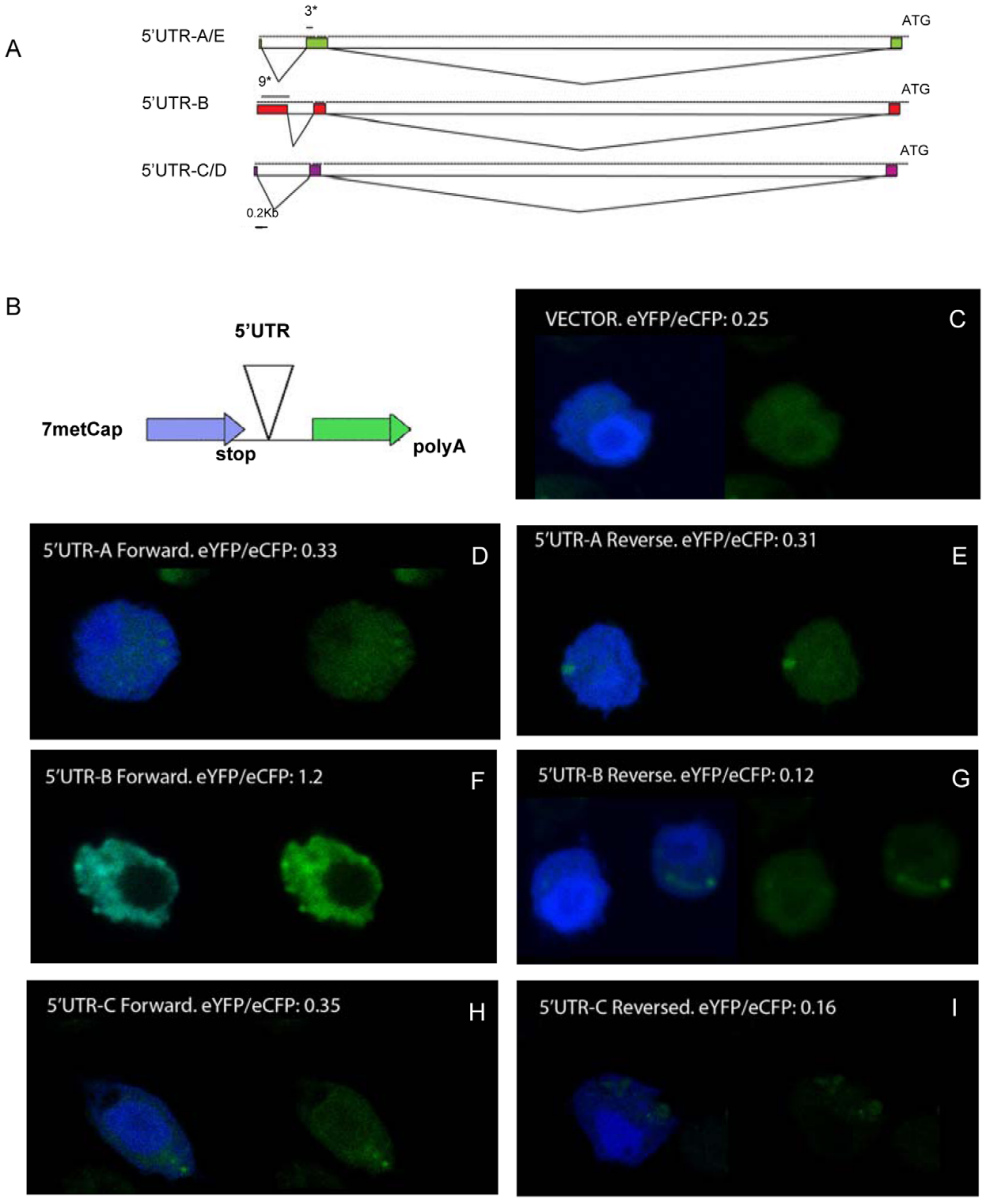
*The dfoxO* 5′UTR contains IRES. (**A**) Diagram of the genomic region of predicted dfoxO's 5′UTRS. 5′UTR-A and -E are similar (5′UTR-E has an additional 20 bp located at the 5′ end). These sequences each contain three upstream ATGs (location marked as 3*). 5′UTR-B is the longest 5′UTR and with 9 upstream ATGs (location marked as 9*). 5′UTR-C and -D are identical and have no upstream ATG. (**B**) Scheme of the di-cistronic reporter construct. Enhanced cyan fluorescent protein (eCFP) in blue and enhanced yellow fluorescent protein (eYFP) in green. (**C–E**) Drosophila S2 cells transfected with eCFP/eYFP di-cistroninc reporter. (**C**) empty vector (**D**) vector with *dfoxO* 5′UTR-A in forward 5′-3′orientation, (**E**) and vector *dfoxO* 5′UTR-A in reverse 3′-5′orientation. (**F**) vector with *dfoxO* 5′UTR-B in forward 5′-3′orientation, (**G**) and vector *dfoxO* 5′UTR-B in reverse 3′-5′orientation. (**H**) vector with *dfoxO* 5′UTR-C in forward 5′-3′orientation, (**I**) and vector *dfoxO* 5′UTR-C in reverse 3′-5′orientation. The ratios eYFP to eCFP fluorescence are shown above the pictures.

Because cap-dependent translation is decreased when cells and animals are fasted, we looked to see if dFoxO proteins are maintained during fasting through a cap-independent translation mechanism such as IRES translation. mRNAs containing IRES have long 5′UTRs that contain multiple upstream AUG sequences [26]. The *dfoxO* gene has four unique 5′UTRs; all are relatively long, being greater than the average size of 256 bp in Drosophila [27], and three *dfoxO* 5′UTRs contain internal AUGs (Fig. 2A). 5′UTR-A and -E are similar (5′UTR-E has an additional 20 bp located at the 5′ end). These sequences each contain three upstream ATGs. 5′UTR-B is the longest 5′UTR with 9 upstream ATGs. 5′UTR-C and -D are identical and have no upstream ATG (Fig. 2A).

We cloned *dfoxO* 5′UTR A, B and C between protein-encoding reporter sequences of a di-cistronic construct [28] (Fig. 2D–I). In this construct (Fig. 2B), cap-dependent translation is reported by enhanced cyan fluorescent protein (eCFP). Translation of the second cistron, reported by enhanced yellow fluorescent protein (eYFP), is inefficient unless the cloned UTR contains a functional IRES. Each of these vectors was transfected into Drosophila S2 cells. In cells maintained on nutrient deplete serum free media, only 5′ UTR-B, the longest of *dfoxO* 5′ UTRs, which contains 9 upstream AUGs (Fig 2A), was able to initiate IRES-mediated translation of eYFP (compare Fig 2D, 2H with 2F). As controls we transfected empty vectors or vectors with the 5′UTRs in reverse orientation. Neither of these controls showed IRES activity (Fig. 2C,E,G,I).

### *dfoxO*-IRES and *dInR*-IRES translation is active when fasting represses cap-dependent translation in insects cells

**Figure 3:**
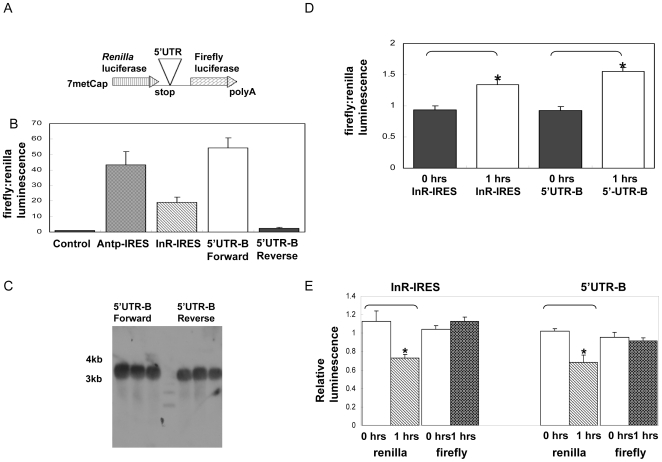
Translation from *dfoxO*-IRES and *dInR*-IRES when fasting represses cap-dependent translation of S2 cells. (**A**) Scheme of the di-cistronic reporter construct. Translation from 5′cap is quantified by renilla luminescence. Translation from the IRES of the same cells is quantified from firefly luminescence. (**B**) Ratio of firefly-to renilla luminescence of *dInR-IRES*, *Antp-IRES* and *dfoxO*-5′UTR-B in forward and reverse orientation. (**C**) Northern analysis from S2 cells transfected with the di-cistronic renilla-fifefly luciferase reporter with *dfoxO*-5′UTRB firefly luciferase inserted in forward and reverse orientation. Transfections were done in triplicate. The probe used is specific to firefly luciferase. (**D**) Ratio of firefly to renilla luminescence of dInR-IRES and dFoxO-5′UTR transfected cells before (0 hours) and after fasting (1 hour). (**E**) Renilla and firefly luminescence of *dInR*-IRES and *dfoxO*-5′UTR in transfected cells before fasting (0 hours) and after fasting (1 hour). Data are shown as the mean +1SD for duplicates determinations. *, P<0.05 versus 0 hrs of fasting.

The level of dFoxO protein may be preserved in fasted cells if IRES mediated translation is active when cap-dependent translation is repressed. To study *dfoxO*-IRES mediated translation we cloned dfoxO 5′UTR-B in a second dicistronic construct in which cap-dependent translation is reported by renilla luciferase, while IRES initiated translation is reported by firefly luciferase [12] (Fig. 3A). Cells transfected with the renilla/firefly vector showed similar results to the 5′UTR-B cloned in the eCFP/eYFP vector, and permitted quantitative measurement based on luminescence. Again, as a control, *dfoxO* IRES translation was inactive with the 5′UTR inserted in reverse orientation (Fig. 3B). The efficiency of *dfoxO* IRES-mediated translation was similar to that of other established Drosophila IRES: *Antennapedia* (*Antp*) and (*dInR*) (Fig. 3B). Northern blot analysis showed that mRNAs from cells transfected with the renilla/firefly *dfoxO* 5′UTR-B inserted in forward orientation were similar in size to those with the *dfoxO* 5′UTR-B in reverse orientation (Fig. 3C).

We starved Drosophila S2 cells transfected with *dfoxO* 5′UTR inserted in the di-cistronic renilla/firefly construct (Fig. 3D). S2 cells maintained in serum free media are more sensitive to stress than those in complete serum. To avoid cell death, we first determined the maximum time of starvation at which cell viability was maintained. Cells were starved in 1× PBS for 0.5, 1, 2, 4 and 6 hours. 1 hour of starvation did not affect cell viability as measured by trypan blue staining (data not shown).

Cells starved for 1 hour in PBS have increased *dfoxO*-IRES-mediated translation (firefly) relative to cap-dependent translation (renilla) (Fig 3D). The increased ratio is due to a reduced expression of renilla luciferase while the *dfoxO*-IRES dependent translation remained constant (Fig 3E). To test if the IRES of dInR shared these behaviors, we studied *dInR*-IRES translation in starved S2 cells transfected with a *dInR*-IRES renilla/firefly di-cistronic construct. Similar to *dfoxO*, the ratio of firefly to renilla luciferase expression increased (Fig. 3C) in fasted cells due to a decrease in cap-dependent translation (Fig. 3E).

## Discussion

FoxO protein is tightly regulated by post-translational modifications including phosphorylation, ubiquitination and acetylation. These regulate its sub-cellular localization, protein stability, DNA binding properties and transcriptional activity [25]. Here we suggest an additional level of control based on the regulation of the initiation of dFoxO translation by IRES.

IRES mediated translation provides yeast cells with proteins needed to cope with starvation [5]. Our data show that under starvation, S2 cells decrease cap-dependent translation as seen by a decrease in the expression of the reporter renilla luciferase, while the reporter for *dfoxO* IRES dependent translation remains unchanged. This finding suggests that the regulation of dFoxO translation by IRES may help retain essential cellular functions during Drosophila fasting.

dFoxO is required for optimal survival during fasting and dFoxO protein remains constant when the level of total protein decreases in nutrient deprived flies. Based on analysis with S2 cells, this retention of dFoxO protein may in part be explained by IRES mediated translation when cap-dependent translation is otherwise depressed. Previous work has shown that dFoxO protein is dephosphorylated and active in nutrient deprived S2 cells [15], leading to the up-regulation of genes needed for fasting adaptation, such as the translational repressor 4E-BP [19], [20], [21] as well as the insulin receptor itself [15]. Interestingly, our data suggest that in fasted flies, a relative increase in dFoxO protein rather than nuclear translocation is responsible for dFoxO activation (Fig 1C) [25]. Notably, dInR also harbors an IRES [12], and we show that dInR-IRES mediated translation is active in starved S2 cells (Fig. 3). IRES appear to maintain translation of both dInR and dFoxO during nutrient stress in vitro, suggesting that the homeostatic reciprocal relationship between dInR and dFoxO includes not only a mechanism to transcribe the insulin receptor but also a system to ensure production of both dInR and dFoxO proteins. Such a system may also occur in mammals because the concentration of InR protein has been shown to increase substantially in starved HepG2 cells [29]. Our work suggests that the maintenance of InR and FoxO during fasting may ensure there is sufficient FoxO to transcribe genes that are essential for cells to cope with limited nutrients and to increase the potential insulin sensitivity of cells in anticipation of the time when nutrients are again available.

## Materials and Methods

### Drosophila strains and fasting survival assay

The *dfoxO* mutants *dfoxO^21^* and *dfoxO^w24^* are described in [30] and [31] respectively. Because the *dfoxO^21^* original stock is homozygote lethal but *dfoxO* null mutants are homozygous viable [24], [30], we recombined *dfoxO^21^* with *yw* to eliminate second site mutations and generate homozygous viable lines. Afterward, *dfoxO^2^* ^1^ and *dfoxO^w24^* stocks were backcrossed to *yw* for four generations. In all trials *yw* were used as controls. To assess survival in fasting adults, newly eclosed adult flies were collected within 24 hour. Adults were aged for 4 days under controlled conditions (25°C, 40% relative humidity and 12 h light∶12 h dark) on normal media (11% sugar, 2.5% autolyzed yeast, 5.2% cornmeal, agar 0.79% w/v in water and 0.2% tegosep -methyl 4-hydroxybenzoate, from Sigma- St. Louis, MO, USA-). At the age of 5 days, 10 females per vial and 10 vials per genotype were transfered into media for fasting (0.8% agarose w/v in PBS). Dead flies were scored in each vial at 6 hour intervals. Differences among survivorship were assessed by the log rank test [32].

### Western Blotting and Cell fractionation

Lysates were generated separately from decapitated bodies of 120 two to four day-old females. Prior to analysis flies were maintained on normal diet or fasting diet for 48 hours. Cytoplasmic and nuclear extracts were prepared from lysed tissues by using the Active Motif Nuclear Extract Kit (Carlsbad, CA. USA, Cat #40010). Total protein concentration was determined using the BCA Protein Assay Kit (Thermo Scientific, Rockford, IL, USA.) following the manufacturers' protocols. For Western blot analysis, 4 µg of total protein per lane were separated using NuPAGE 4–12% Bis-Tris Gel (Invitrogen, Carlsbad, CA, USA.) and electrotransferred to PVDF membrane. Fasted and fed samples were loaded in the same gel. 3 different gels were blotted independently for dFoxO, Lamin and Hsp90. Each membrane was exposed at different times dependently on the antibody- specific antigen recognition.

Antibodies were guinea pig anti-dFoxO (gift from H. Broihier, Case Western Reserve University), mouse anti-Lamin DMO (ADL84.12, Developmental Studies Hybridoma Bank, University of Iowa, Iowa, USA), and rabbit anti-Hsp90 (SPA-846 Stressgen, Ann Arbor, Michigan, USA). Following blotting, the PVDF membranes were stained with Coomassie to verify that equal amounts of protein were transferred. Each experiment was repeated twice.

### Quantitative PCR

cDNA was prepared from 25 decapitated bodies from each of three biological samples. Each sample used six to eight day-old *yw* females that had previously been maintained on a normal or fasting diet for 48 hours. For qPCR, three technical replicates were generated per biological sample. *dfoxO* mRNA was amplified by the primer pair caatctcgagtgcaatgtcgagga (forward) and ccctgagcatcagcaacattagca (reverse) using Superscript III Platinum SYBR Green One-Step qRT-PCR with ROX (cat. 11746-500, Invitrogen, Carlsbad, CA, USA.) on an ABI 7300 System and standardized against the *RPL32* mRNA (forward primer: aatgatgtgcgagtgccgag, reverse primer: caatggtgctgctatcccaatc).

### Cloning *dfoxO* 5′UTR

5′UTR sequences of the *dfoxO* transcript are described in www.flybase.org (Schematic representation in Fig. 2A). To generate the eCFP/eYFP dicistronic construct, the 5′UTR-A, B and C of *dfoxO* was amplified with the primers aaccatggctagaaagcagttaacgagtagtc (forward) and tttttccatggggtagaaaagttcgttgtgcagt (reverse) from clones LD14524, LD19191 and LD05569 (Berkeley Drosophila Genome Project) respectively. The amplified fragment was subcloned into pNEX3-eCFP –eYFP (provided by John R. Dyer and Wayne S. Sossin [28]) using Nco1sites. The eCFP-5′UTR-eYFP fragment was then PCR amplified with the primers acgcggaattcatggtgagcaagggcgaggagctg (forward) and ttgggcccggtaccttacttgtacagctcgtccatgccg (reverse), and subsequently subcloned into the pAc5.1/V5-HisA vector (Invitrogen) using the EcoRI (5′) and ApaI (3′) sites. To clone this *dfoxO* 5′UTR into the renilla/firefly di-cistronic construct, the 5′UTR was amplified as before and cloned into the pMARL vector [12] using Nco1 sites.

Renilla-InR-5′UTR-firefly and renilla-Antennapedia-5′UTR-firefly constructs were kindly provided by Michael Marr and Robert Tjian [12].

### S2 cell transient transfection

The di-cistronic constructs were transfected into Drosophila S2 cells in serum free media Express Five® SFM (Invitrogen) using Effectene Transfection Reagent (Quiagen, Valencia, CA, USA) following the manufacturer's instructions. Images (objective oil immersion 63×; numeral aperture 1.4) were collected using a Leica TCS SP2 AOBS Confocal Laser Scanning Microscope, 48 hours post-transfection. Samples were mounted in ProLong® Gold antifade reagents (Invitrogen, Carlsbad, CA, USA). Fluorescence was analyzed using the Leica Confocal Software Lite Version. Luciferase activity was measured by using the Dual-Glo™ Luciferase Assay System (Promega) with a SpectraMax M5 microplate reader and SoftMax Pro 4.8. software (Molecular Devices, Sunnyvale, CA, USA.) following the manufacturer's instructions. For starvation assays, 48 hours post-transfection, an equal number of cells from a transfection event were transferred to 1× PBS or media for one hour, and measured for luciferase activity. Three technical replicates were used per treatment. Each transfection was repeated three times. The experiment was repeated twice.

### Northern blot analyses

Transfections were performed as described above. RNA was extracted with TRIzol® Reagent following the manufacturer's instructions (Invitrogen, Carlsbad, CA, USA.). Northern blots were conducted with the NorthernMax® Kit following manufacturer's instructions (Ambion, Austin, TX, USA.). Purified RNA (15 µg per lane) was electrophoresed, blotted onto a nylon membrane and probed for firefly luciferase. ^32^P-labelled probe was synthesized by random primer using the DECAprime™ II kit from Ambiom (Austin, TX, U.S.A) following the manufacturer's instructions.

## Statistical analyses

Data are shown as the mean and standard deviation. An unpaired t-test was used for all comparisons. Differences were considered significant at p<0.05. Differences among survivorship were assessed by the log rank test [32].
